# The Central Negative Regulator of Flooding Tolerance, the PROTEOLYSIS 6 Branch of the N-degron Pathway, Adversely Modulates Salinity Tolerance in *Arabidopsis*

**DOI:** 10.3390/plants9111415

**Published:** 2020-10-23

**Authors:** Suman Lamichhane, Jasper B. Alpuerto, Abigail Han, Takeshi Fukao

**Affiliations:** 1 School of Plant and Environmental Sciences, Virginia Tech, Blacksburg, VA 24061, USA; sumanl7@vt.edu (S.L.); Jasper.Alpuerto@ag.tamu.edu (J.B.A.); ahan@vt.edu (A.H.); 2 Texas A & M Agrilife Research, Beaumont, TX 77713, USA; 3 Department of Bioscience and Biotechnology, Fukui Prefectural University, Eiheiji, Fukui 910-1195, Japan

**Keywords:** *Arabidopsis thaliana*, brassinosteroids, ethylene, N-degron pathway, *PRT6*, salinity

## Abstract

Seawater intrusion in coastal regions and waterlogging in salinized lands are serious constraints that reduce crop productivity under changing climate scenarios. Under these conditions, plants encounter flooding and salinity concurrently or sequentially. Identification and characterization of genes and pathways associated with both flooding and salinity adaptation are critical steps for the simultaneous improvement of plant tolerance to these stresses. The PROTEOLYSIS 6 (PRT6) branch of the N-degron pathway is a well-characterized process that negatively regulates flooding tolerance in plants. Here, we determined the role of the PRT6/N-degron pathway in salinity tolerance in *Arabidopsis*. This study demonstrates that the *prt6* mutation enhances salinity tolerance at the germination, seedling, and adult plant stages. Maintenance of chlorophyll content and root growth under high salt in the *prt6* mutant was linked with the restricted accumulation of sodium ions (Na^+^) in shoots and roots of the mutant genotype. The *prt6* mutation also stimulated mRNA accumulation of key transcription factors in ABA-dependent and independent pathways of osmotic/salinity tolerance, accompanied by the prominent expression of their downstream genes. Furthermore, the *prt6* mutant displayed increased sensitivity to ethylene and brassinosteroids, which can suppress Na^+^ uptake and promote the expression of stress-responsive genes. This study provides genetic evidence that both salinity and flooding tolerance is coordinated through a common regulatory pathway in *Arabidopsis*.

## 1. Introduction

Most commercially important plants are susceptible to high salt levels. Therefore, soil salinization is a serious constraint that threatens food security worldwide. Currently, more than 6% of the total land surface and 20% of the total irrigated areas are affected by excess salt, a condition that is increasingly widespread [1]. To meet growing demands for plant-based products at the global level, it is imperative to improve salinity tolerance in major crops.

Plants encounter multiple abiotic stresses simultaneously or sequentially in an agricultural or natural environment. In the context of salinity stress, plants can be exposed to excess salt and water as a consequence of flooding in salinized areas and seawater intrusion in coastal regions. Importantly, much of the world’s saline land is subjected to flooding because of shallow water tables and low infiltration of surface water [2]. It has been predicted that a rise in the sea level and elevated frequency of storm surges will increase seawater inundation in low-lying grasslands and croplands, leading to serious agricultural losses in coastal zones [3,4,5]. For these reasons, there is an urgent need for the development of new crop varieties with enhanced tolerance to both salinity and flooding.

Identification and functional characterization of genes and pathways associated with adaptation to both salinity and flooding are critical steps for the simultaneous improvement of plant tolerance to these stresses. In this study, we evaluated whether a well-characterized signaling pathway involved in flooding tolerance, the PROTEOLYSIS 6 (PRT6) branch of the N-degron pathway, regulates adaptation to high salt. The N-degron pathway is an enzymatic cascade that determines the half-life of protein substrates depending on the identity of their N-terminal residues [6,7]. In the PRT6 branch of the N-degron pathway, PRT6, an E3 ubiquitin ligase, is the last enzyme that recognizes the specific N-terminal residues of its substrates, which are subsequently ubiquitinated and transported into the 26S proteasome for degradation [8].

Diverse biological roles of the PRT6/N-degron pathway have been revealed through genetic analysis of loss-of-function mutants of *prt6* and other enzymes of this pathway. Such roles include the regulation of seed dormancy and germination, seedling establishment, leaf and root development, photomorphogenesis, leaf senescence, and disease resistance as well as flooding/low oxygen tolerance [9,10,11,12,13,14,15,16,17,18,19,20]. Genetic evidence obtained from these studies suggests that proper modulation of regulatory protein levels via the PRT6/N-degron pathway coordinates these traits. Nevertheless, only a limited number of the PRT6/N-degron substrates have been identified; the most characterized of these being the group VII Ethylene Response Factor (ERF-VII) transcription factors.

The *Arabidopsis* genome encodes five *ERF-Vll* genes, all of which play a pivotal role in the expression of core hypoxia-responsive genes and tolerance to flooding and low oxygen [21,22]. The half-life of these transcription factor proteins is controlled by sequential reactions in the PRT6/N-degron pathway. All ERF-VII transcription factors contain a highly conserved N-terminal motif that is initiated with methionine (Met) and cysteine (Cys) residues [12,13]. First, the N-terminal Met is cleaved by methionine aminopeptidase. Next, the exposed Cys is oxidized by plant cysteine oxidases (PCOs) in an oxygen and nitric oxide-dependent manner [14,15]. The oxidized Cys is then arginylated by arginyl-tRNA transferases. Finally, the N-terminally modified ERF-VII proteins are ubiquitinated by PRT6, which are targeted for proteasomal degradation [8]. Due to the oxygen requirement for PCOs, low oxygen and flooding allow ERF-VII proteins to escape from this proteolysis pathway, thereby triggering the expression of hypoxia-responsive genes. Consistently, disruption of the PRT6/N-degron pathway in *ate1ate2* and *prt6* mutants promotes transcript accumulation of core hypoxia-responsive genes and confers tolerance to oxygen deprivation and submergence [12,15]. It was predicted that the PRT6/N-degron pathway catalyzed substrates other than ERF-VIIs [23]. In fact, a polycomb repressive complex 2 subunit VERNALIZATION 2 (VRN2) and a small leucine zipper-containing protein LITTLE ZIPPER 2 (ZPR2) were recently identified as new N-degron targets in *Arabidopsis* [24,25].

In the present study, we assessed the role of the PRT6/N-degron pathway, a key regulatory process of flooding tolerance, in adaptation to salinity stress in *Arabidopsis* through physiological and molecular characterization of the *prt6-1* mutant. Recently, it was reported that the *prt6* mutation enhances seedling survival under high salt [26]. However, the influence of the mutated gene in salt tolerance at other developmental stages and its adaptation mechanisms remain obscure. Here, we investigated the contribution of the *prt6* mutation to salinity tolerance and relevant traits in *Arabidopsis* at various growth stages. Detailed time-course and dose-response analyses uncovered a negative role of the PRT6/N-degron pathway in transcriptional, hormonal, and physiological adaptations to excess salt. This study provides genetic evidence that both flooding and salinity tolerance is coordinated through a common regulatory pathway in *Arabidopsis.*

## 2. Results

### 2.1. The prt6 Mutation Enhances Salinity Tolerance at Various Developmental Stages

**Figure 1:**
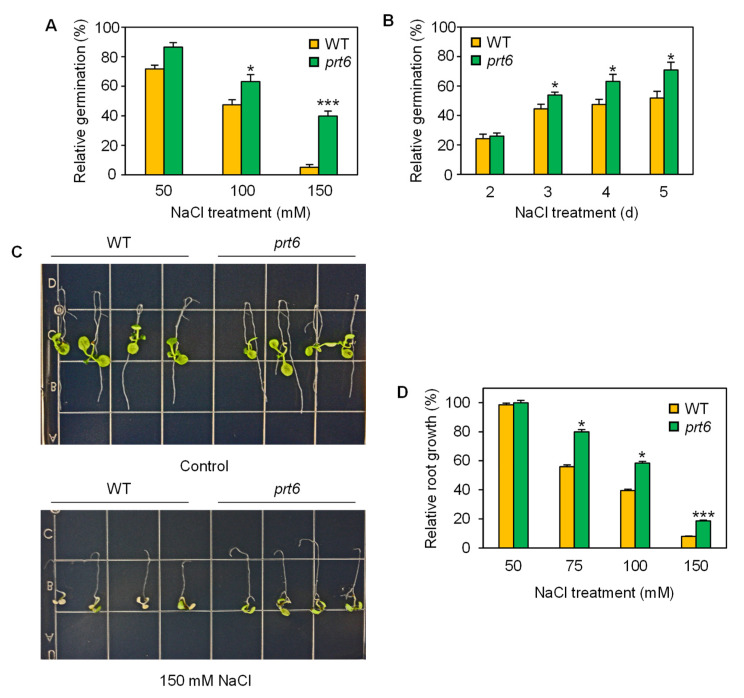
Loss-of-function mutation of *prt6* enhanced tolerance to moderate (sublethal) salinity at the seed germination and seedling stages. (**A**) Dose-response of wild-type (WT) and *prt6* seeds to NaCl during seed germination. Seeds were incubated under sublethal levels of NaCl (≤150 mM) for 4 d. (**B**) Time-course observation of seed germination in WT and *prt6* seeds under sublethal salinity. Seeds were incubated under 100 mM NaCl for up to 5 d. (**C**) Photos of WT and *prt6* seedlings that were grown upside down under non-stress or moderate salinity. Four-day-old seedlings grown under non-stress conditions were transferred on half-strength MS plates containing 0 or 150 mM NaCl and incubated upside down for 4 d. (**D**) Dose-response of WT and *prt6* root growth to moderate salinity. Four-day-old seedlings were grown vertically on half-strength MS plates containing a range of NaCl concentrations for 4 d. Relative germination and root growth in (**A**), (**B**), and (**D**) were calculated by comparison to non-stressed WT or *prt6*. Data represent means ± SE [n = 3 in (**A**,**B**); n = 24 in (**D**)]. Asterisks indicate significant difference between WT and *prt6* (* *p* < 0.05; *** *p* < 0.001).

To determine the role of the *prt6* mutant in salinity tolerance at different growth stages, we performed various stress tolerance evaluations. First, we assessed seed germination performance under moderate (sub-lethal) salinity (≤150 mM NaCl) (Figure 1A). Salinity stress reduced seed germination of both wild-type (WT) and mutant genotypes in a dose-dependent manner, but *prt6* seeds germinated more vigorously at 100 and 150 mM NaCl. Consistently, the time-course observation demonstrated that *prt6* seeds germinated more readily than WT on days 3, 4, and 5 (Figure 1B).

Next, we determined the ability of the *prt6* mutant in root elongation under moderate salinity at the seedling stage. Well-established root bending assays [27,28] were performed to demonstrate the effect of *prt6* mutation on root elongation under salinity stress. In these assays, WT and *prt6* seedlings displayed similar root elongation under control conditions, whereas roots of *prt6* seedlings grew more vigorously than WT under salinity (Figure 1C). Quantitative assays of root elongation further supported that the *prt6* mutation contributes to maintained root growth under moderate salinity (75–150 mM NaCl) (Figure 1D).

**Figure 2:**
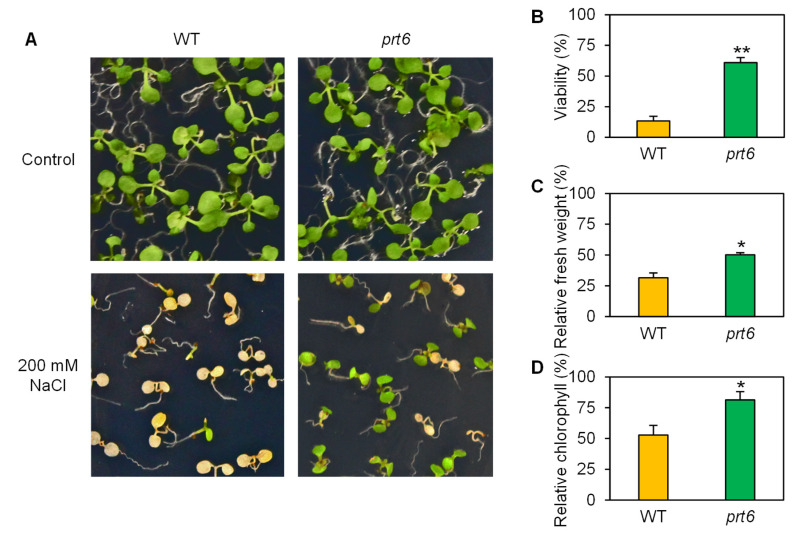
The *prt6* mutation increased seedling viability under high salinity. (**A**) Photos of wild-type (WT) and *prt6* mutant seedlings exposed to non-stress or high salinity conditions. Four-day-old seedlings were transferred onto half-strength MS media containing 0 or 200 mM NaCl and grown for 4 d. Seedling viability (**B**), relative fresh weight (**C**), and relative chlorophyll content (**D**) of WT and *prt6* seedlings under high salinity. Seedlings exposed to 0 or 200 mM NaCl as described in (**A**) were used for data collection in (**B**–**D**). Relative fresh weight and chlorophyll were calculated by comparison to non-stressed WT or *prt6*. Data represent means ± SE [n = 3 in (**B**–**D**)]. Asterisks indicate significant difference between WT and *prt6* (* *p* < 0.05; ** *p* < 0.01).

We also evaluated whether the *prt6* mutation affects seedling viability under high (lethal) salinity. WT and *prt6* seedlings showed identical growth under control conditions (Figure 2A). When exposed to high salinity (200 mM NaCl), more than 90% of WT seedling died, but the majority (>60%) of *prt6* seedlings were viable (Figure 2A,B). Correspondingly, seedling biomass and leaf chlorophyll content were significantly higher in the *prt6* mutant than WT under high salinity (Figure 2C,D).

**Figure 3:**
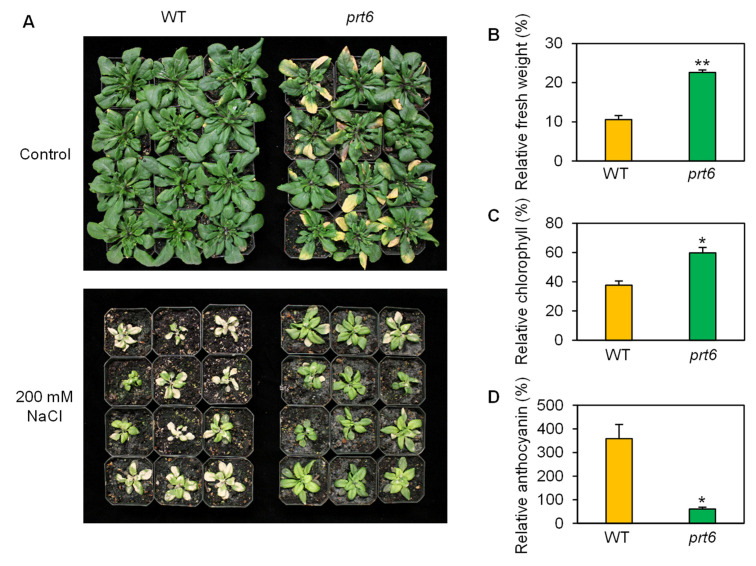
The *prt6* mutation increased adult-plant tolerance to high salinity. (**A**) Photos of wild-type (WT) and *prt6* plants exposed to non-stress or high salinity. Plants were grown in pots under regular growth conditions for 21 d and then irrigated with fertilized water containing 0 or 200 mM NaCl for 12 d. Relative fresh weight (**B**), chlorophyll (**C**), and anthocyanin (**D**) of WT and *prt6* plants exposed to salinity. These relative values were calculated by comparison to non-stressed WT or *prt6*. Data represent means ± SE [n = 12 in (**B**); n = 3 in (**C**,**D**)]. Asterisks indicate significant difference between WT and *prt6* (* *p* < 0.05; ** *p* < 0.01).

Finally, we analyzed adult-plant tolerance to high salinity. Under control conditions, *prt6* plants displayed early senescence, relative to WT (Figure 3A). However, *prt6* plants had more green leaves than WT under high salt. Consistent with the phenotypic observation, aboveground biomass and chlorophyll content were significantly higher in the *prt6* mutant under salt stress (Figure 3C,D). In contrast with the chlorophyll data, anthocyanin was highly accumulated in WT plants (Figure 3D), indicating that WT suffered from more severe salinity damage. Taken together, the qualitative and quantitative data presented here demonstrated that loss-of-function mutation of *prt6* increases salinity tolerance at the seed germination, seedling, and adult-plant stages.

### 2.2. The prt6 Mutant Plants Show Restricted Accumulation of Sodium Ions (Na^+^) in Roots and Shoots

**Figure 4:**
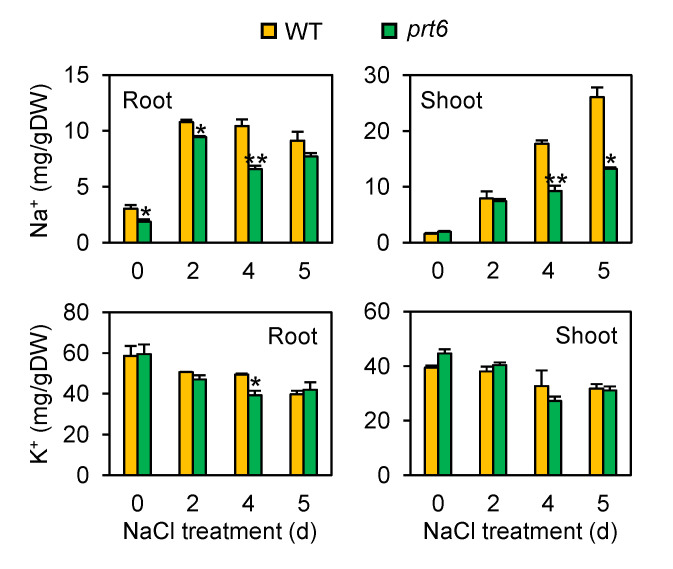
The *prt6* mutation restricted accumulation of Na^+^ in shoots and roots of *Arabidopsis* plants under salinity. Wild-type (WT) and *prt6* plants were grown in Turface under regular growth conditions for 21 d and then irrigated with fertilized water containing 150 mM NaCl for up to 5 d. Shoot and root samples were harvested at the specified time points, washed thoroughly, and subjected to Na^+^ and K^+^ assays. Data represent means ± SE (n = 3). Asterisks indicate significant difference between WT and *prt6* (* *p* < 0.05; ** *p* < 0.01).

Salinity-mediated reductions in chlorophyll content and root growth were less severe in the *prt6* mutant than WT. We hypothesized that these results are attributed to a restricted accumulation of Na^+^ in the mutant line. To test this, we monitored the abundance of Na^+^ in root and shoot tissues of plants exposed to salinity stress. This analysis revealed that the *prt6* mutant contained a lower amount of Na^+^ in both roots and shoots (Figure 4). In shoots, the effect of this mutant allele was still significant on day 5, while this effect was not observed in roots at that time. These results suggest that the *prt6* mutation contributes to the suppression of root-to-shoot Na^+^ transport as well as Na^+^ uptake into roots. Unlike Na^+^ concentrations, the influence of the mutant allele in K^+^ accumulation was minimal or not observed in roots and shoots (Figure 4).

### 2.3. The prt6 Mutation Augments Responsiveness to Ethylene and Brassinosteroids

**Figure 5:**
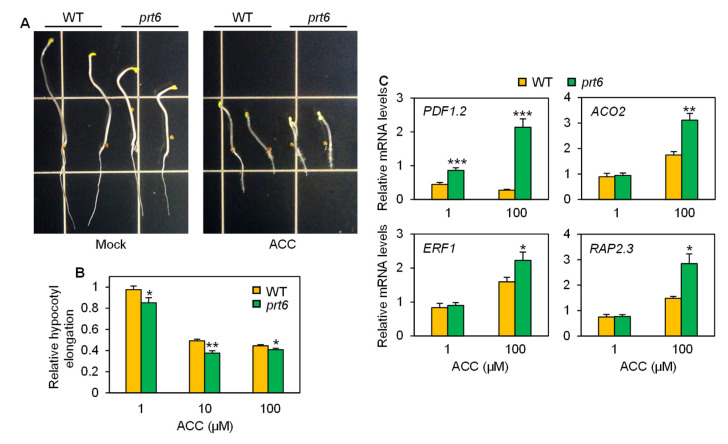
The *prt6* mutation enhanced ethylene responsiveness. (**A**) Photos of wild-type (WT) and *prt6* seedlings that were grown on half-strength MS media containing 0 (mock) or 10 µM ACC for 5 d in the dark. (**B**) Relative hypocotyl elongation of WT and *prt6* seedlings treated with ACC. Seeds were incubated on half-strength MS plates containing a range of ACC concentrations for 5 d in the dark. Hypocotyl length was measured using Image J software. Data represent means ± SE (n = 24). (**C**) Relative mRNA levels of ethylene-responsive genes. Ten-day-old seedlings were treated with 1 or 100 µM ACC for 6 h. Relative hypocotyl elongation and mRNA levels were calculated by comparison to the corresponding genotype (WT or *prt6*) under mock (0 mM ACC) conditions. Data represent means ± SE (n = 3). Asterisks indicate significant difference between WT and *prt6* (* *p* < 0.05; ** *p* < 0.01, *** *p* < 0.001).

Ethylene and brassinosteroids (BR) are positive regulators for salinity tolerance in plants [29,30,31,32]. To discern the capability of the *prt6* mutant in regulating sensitivity to these hormones, we performed hormone response assays. When incubated under constant darkness, *prt6* mutant seedlings displayed moderate triple response phenotypes even without 1-aminocyclopropane-1-carboxylic acid (ACC), an immediate precursor of ethylene (Figure 5A). When ACC was supplied, more severe restriction of hypocotyl elongation and enhancement of apical hook formation were observed in the mutant seedlings. Dose-response analysis further supported that ACC-mediated reductions in hypocotyl growth were more significant in the *prt6* mutant at all ACC concentrations tested (Figure 5B). We also evaluated the effect of the mutant allele on mRNA accumulation of ethylene-responsive genes. All the four representative marker genes, *PLANT DEFENSIN 1.2* (*PDF1.2*), *ACC OXIDASE 2* (*ACO2*), *ERF1*, and *RELATED TO AP2.3* (*RAP2.3*) [33,34], were highly induced in response to ACC in the *prt6* mutant, compared to WT (Figure 5C). These results indicate that the *prt6* mutation leads to increased responsiveness to ethylene at the molecular and physiological levels. 

**Figure 6:**
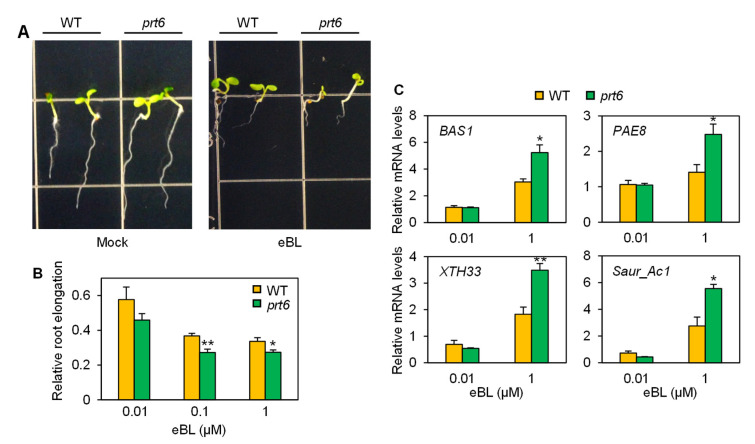
The *prt6* mutation augmented brassinosteroid responsiveness. (**A**) Photos of WT (wild-type) and *prt6* seedlings that were grown under mock (0.02% ethanol) or 24-epibrassinolide (eBL; 1 µM in 0.02% ethanol) for 5 d. (**B**) Relative root elongation of WT and *prt6* seedlings treated with eBL. Seeds were incubated on half-strength MS plates containing a range of eBL concentrations for 5 d. Root length was measured using Image J software. Data represent means ± SE (n = 18). (**C**) Relative mRNA levels of brassinosteroid-responsive genes. Ten-day-old seedlings were treated with eBL (0.01 or 1 µM in 0.02% ethanol) for 6 h. Relative root elongation and mRNA levels were calculated by comparison to the corresponding genotype (WT or *prt6*) under mock (0.02% ethanol) conditions. Data represent means ± SE (n = 3). Asterisks indicate significant difference between WT and *prt6* (* *p* < 0.05; ** *p* < 0.01).

We also examined the impact of the *prt6* mutation on BR sensitivity. Both WT and *prt6* seedlings grew similarly under mock (0.02% ethanol) conditions (Figure 6A). Application of 24-epibrassinolide (eBL), a bioactive BR, considerably limited root growth in the two genotypes, with a more severe reduction in the mutant seedlings. Dose-dependent analysis verified that root elongation was inhibited to a greater degree by 0.1 and 1 µM eBL in the *prt6* mutant than WT. BR responsiveness in WT and the *prt6* mutant was further evaluated by expression analysis of BR-responsive genes. All the four marker genes, *PHYB ACTIVATION TAGGED SUPPRESSOR* 1 *(BAS1*), *PECTIN ACETYLESTERASE 8* (*PAE8*), *XYLOGLUCAN: XYLOGLUCOSYL TRANSFERASE 33* (*XTH33*), and *SMALL AUXIN UP RNA_Ac1* (*Saur_Ac1*) [35,36], were expressed more abundantly in the mutant line than WT when treated with 1 µM eBL (Figure 6C). Altogether, these data demonstrated that the *prt6* mutation contributes to amplified sensitivity to BR.

### 2.4. The prt6 Mutation Activates ABA-Dependent and Independent Pathways Involved in Salinity/Osmotic Stress Tolerance

**Figure 7:**
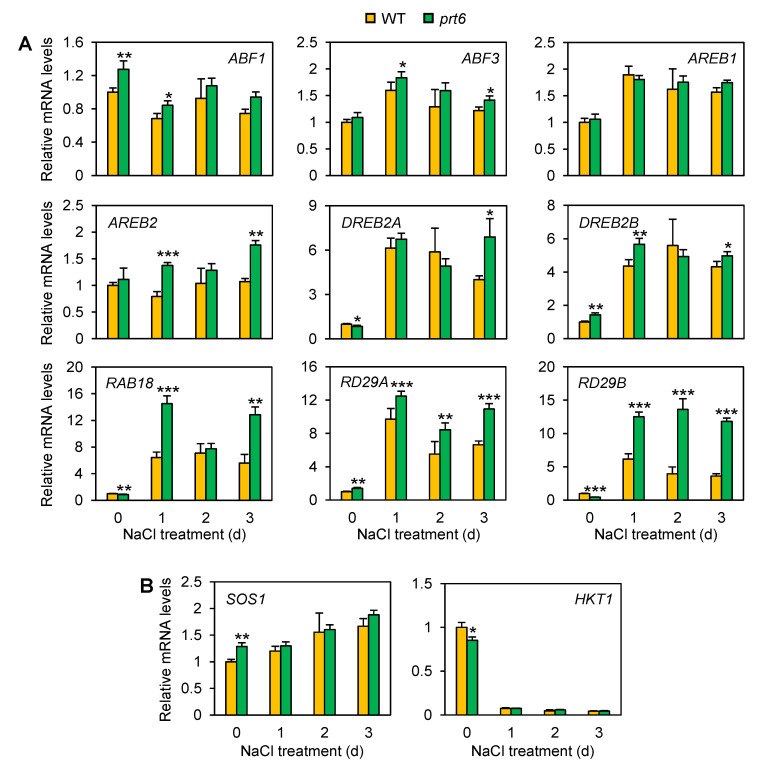
The *prt6* mutation induced mRNA accumulation of genes associated with salinity tolerance. (**A**) Relative mRNA levels of transcription factors involved in ABA-dependent (*AREB*/*ABFs*) and independent (*DREB2s*) pathways of salinity/osmotic stress tolerance and their downstream genes, dehydrins. (**B**) Relative mRNA levels of sodium transporter genes. Ten-day-old seedlings were treated with 150 mM NaCl for up to 3 d and subjected to qRT-PCR analysis. Data represent means ± SE (n = 3). Asterisks indicate significant difference between WT and *prt6* (* *p* < 0.05; ** *p* < 0.01, *** *p* < 0.001).

It has been widely accepted that adaptive responses to drought and salinity are regulated through ABA-dependent and independent pathways at the transcriptional level [37,38]. To determine if the *prt6* mutation alters mRNA accumulation of transcription factors involved in ABA-dependent and independent pathways, the transcript levels of representative genes were monitored in the *prt6* and WT seedlings by qRT-PCR (Figure 7A). Of the four *ABSCISIC ACID-RESPONSIVE ELEMENT-BINDING PROTEINS/FACTORS* (*AREB*/*ABF*s) in ABA-dependent pathways, *ABF1*, *ABF3*, and *AREB2* mRNAs were more abundantly accumulated in the *prt6* mutant than WT in at least one time point under salinity stress. Similarly, *DEHYDRATION-RESPONSIVE ELEMENT-BINDING FACTOR 2A* (*DREB2A*) and *DREB2B*, representative transcription factors in ABA-independent pathways, were more highly induced by salinity stress in the mutant line relative to WT in at least one time point. In contrast with these results, the level of *DREB2A* transcript was higher in WT than the mutant line on day 0, although the difference between the two genotypes was minimal. We also investigated the expression levels of dehydrin genes such as *RESPONSIVE TO ABA 18* (*RAB18*), *RESPONSIVE TO DESICCATION 29A* (*RD29A*), and *RD29B*, direct targets of *AREB*/*ABF*s and *DREB2*s [39,40]. All of these genes were highly induced in response to salinity, with greater expression in the *prt6* mutant during salinity stress (Figure 7A). These results are in accordance with the expression patterns of their upstream transcription factors.

The *prt6* mutant displayed restricted accumulation of Na^+^ in root and shoot tissues (Figure 4). This data raised the question of whether the *prt6* mutation affects the expression of genes associated with Na^+^ transport. To answer this question, we monitored the mRNA accumulation of *SALT OVERLY SENSITIVE 1* (*SOS1*) and *HIGH-AFFINITY K^+^ TRANSPORTER 1* (*HKT1*) by qRT-PCR (Figure 7B). The level of *SOS1* transcript was more abundant in the mutant than WT on day 0, but no significant difference was observed in the two genotypes during salinity stress. The mRNA level of *HKT1* was higher in WT than the mutant line on day 0, but its expression was drastically declined by salinity stress, with no significant difference between the two lines. These results suggest that low accumulation of Na^+^ in mutant roots and shoots is not controlled through transcriptional regulation of these Na^+^ transporters.

### 2.5. The prt6 Mutation Induces ERF-VIIs at the mRNA Accumulation Level under Salinity Stress

**Figure 8:**
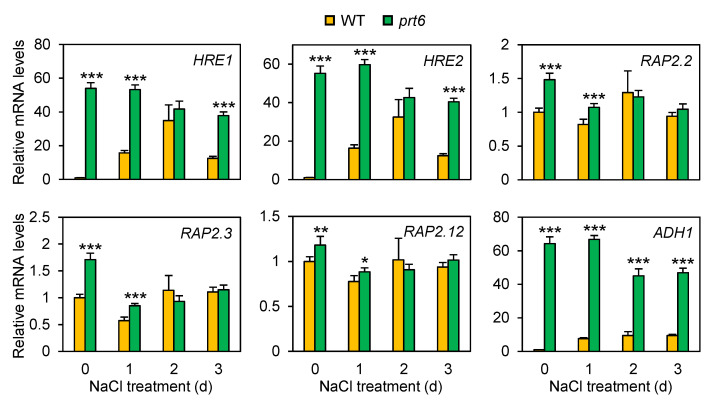
The *prt6* mutation promoted mRNA accumulation of *ERF-VII* genes. Ten-day-old seedlings were treated with 150 mM NaCl for up to 3 d and subjected to qRT-PCR analysis. Data represent means ± SE (n = 3). Asterisks indicate significant difference between WT and *prt6* (* *p* < 0.05; ** *p* < 0.01, *** *p* < 0.001).

*PRT6* encodes the last enzyme in the PRT6/N-degron pathway that regulates the turnover of ERF-VII proteins in *Arabidopsis* [21]. Therefore, a knockout mutation of *prt6* increases the accumulation of ERF-VII proteins [12,13,14]. To determine the influence of the *prt6* mutation in the expression of *ERF-VII*s, we performed qRT-PCR analysis in the *prt6* mutant and WT seedlings. Of the five *ERF-VII* genes, *HYPOXIA RESPONSIVE ERF 1* (*HRE1*) and *HRE2* have been demonstrated to be induced under oxygen deprivation [41,42,43]. Similar to the low oxygen response, these genes were upregulated by salinity stress in WT (Figure 8). In the *prt6* mutant, *HRE1* and *HRE2* were constitutively expressed, and their mRNA level was significantly higher in the mutant than WT under non-stress (day 0) and salinity conditions (days 1 and 3). *RAP2.2*, *RAP2.3*, and *RAP2.12* have not been demonstrated to be responsive to low oxygen [41,43]. Likewise, the transcript abundance of the three *RAP2* genes was not clearly altered under salinity stress in the two genotypes, with higher mRNA accumulation of these genes in the *prt6* mutant. *ALCOHOL DEHYDROGENASE* 1 (*ADH1*) is a downstream gene of most ERF-VIIs; overexpression of *HRE1*, *RAP2.2*, *RAP2.3*, or *RAP2.12* upregulates the expression of *ADH1* even under non-stress conditions [41,43]. Consistent with the *ERF-VII* gene expression data presented here, *ADH1* transcript was highly accumulated in the *prt6* mutant under non-stress and salinity conditions.

## 3. Discussion

Salinity and flooding are closely related abiotic stressors which can occur sequentially or concurrently [2,5]. Therefore, simultaneous improvement of both salinity and flooding tolerance is a desirable trait in crop plants. The PRT6/N-degron pathway is the key signaling process that adversely regulates tolerance to flooding and low oxygen [8,21]. In the present study, we demonstrated that this pathway also plays a negative role in salinity tolerance at the seed germination to adult plant stages through detailed time-course and dose-response observations of the *Arabidopsis prt6* mutant and wild-type plants. Functional characterization of the mutated *prt6* uncovered the transcriptional and hormonal pathways coordinated by the PRT6/N-degron under high salt.

Ethylene is a crucial hormone that regulates plant responses and adaptation to salinity [29,30,32]. For example, exogenous application of an ethylene precursor, ACC, suppressed the accumulation of Na^+^ in *Arabidopsis* roots and reduced membrane damage under excess salt [44]. In the *eto1* mutant that constitutively overproduces ethylene, the Na^+^ concentrations in both stelar cells and xylem sap were reduced under salinity, leading to enhanced plant survival and leaf chlorophyll content [45]. These results indicate that ethylene plays a pivotal role in limiting Na^+^ uptake in roots and transport to shoots under high salt. Besides Na^+^ influx/transport regulation, mutant and transgenic studies revealed that ethylene signaling is necessary for the salt-induced accumulation of dehydrin mRNAs such as *RD29A*, *RD29B*, and *COLD-REGULATED 15A* (*COR15A*) in *Arabidopsis* [46,47]. In the present study, we demonstrated that the *prt6* mutation reduces Na^+^ levels in both roots and shoots (Figure 4) and increases mRNA accumulation of dehydrins including *RD29A* and *RB29B* under salinity (Figure 7). It is anticipated that these adaptive responses enhanced by the *prt6* mutation can be attributed to increased sensitivity to ethylene in the mutant (Figure 5).

BR is another positive regulator for salinity tolerance [31,48,49]. Treatment of barley and canola plants with eBL reduced Na^+^ levels in roots and shoots, contributing to the maintenance of growth under salinity stress [50,51]. Application of eBL also promoted mRNA accumulation of *RD29A* in *Arabidopsis* under excess salt [52], whereas mRNA accumulation of this gene was restricted in a brassinosteroid deficient mutant, *det2* [53]. These results indicate that Na^+^ influx/transport and dehydrin gene expression under salinity stress were coordinated by BR as well as ethylene. It is expected that increased sensitivity to BR in the *prt6* mutant (Figure 6) benefits restricted accumulation of Na^+^ in roots and shoots and stimulated expression of dehydrin genes under high salt (Figure 4 and Figure 7). The PRT6/N-degron pathway is responsible for proteasomal degradation of ERF-VII, VRN2, and ZPR2 proteins [24,25,54], but this pathway may also regulate targeted proteolysis of other proteins. Further investigation is required to uncover how disruption of the PRT6/N-degron pathway enhances plant response to ethylene and BR through stabilization of ERF-VII, VRN2, ZPR2, and other unidentified proteins.

Previous studies showed that mutations in *prt6* increase ABA sensitivity during germination and seedling establishment in *Arabidopsis* [11,19]. Transient expression and chromatin immunoprecipitation (ChIP) analyses demonstrated that RAP2-type ERF-VIIs directly interact with the promoter region of ABA INSENSITIVE 5 (ABI5), a major downstream transcription factor in the ABA signaling pathway, activating its expression [14]. Additionally, GUS staining assays showed that *promABI5::GUS* activity is induced in *prt6* mutant seeds, but not in wild-type seeds. These data indicate that ABA hypersensitivity in *prt6* mutants is caused by enhanced expression of *ABI5* via stabilization of its transcriptional regulators, RAP2-type ERF-VIIs. Our study revealed that the *prt6* mutation increased mRNA accumulation of three *AREB*/*ARF* genes under excess salt (Figure 7). Similar to ABI5, AREB/ABFs serve as downstream transcription factors in the ABA signaling pathway. AREB/ABFs primarily regulate the expression of ABA-responsible genes associated with stress tolerance [55], whereas ABI5 coordinates the expression of ABA-responsive genes related to seed germination and dormancy [56]. It is anticipated that increased mRNA accumulation of *AREB*/*ABF*s in the *prt6* mutant may be attributed to ERF-VII-mediated activation of their respective promoters.

Although many stress-responsive genes are upregulated by ABA, these genes are also induced in an ABA-independent manner [55]. DREB2A and DREB2B are major transcription factors responsible for ABA-independent gene expression. Our study found that the *prt6* mutation increases mRNA accumulation of *DREB2A* and *DREB2B* under high salt, accompanied by high expression of key ABA-dependent transcription factors including *ABF1*, *ABF3*, and *AREB2* (Figure 7). A previous study demonstrated that the expression of *DREB2A* is regulated by direct binding of ABF3, AREB1, and AREB2 to its promoter region under osmotic stress [57]. It is likely that elevated accumulation of *DREB2A* mRNA in the *prt6* mutant results from transcriptional activation mediated by ABF3 and AREB2.

The PRT6/N-end rule pathway regulates the turnover of all five ERF-VII proteins [12,13,14]. Thus, disruption of *prt6*, which encodes an essential enzyme of the proteolysis pathway, leads to overaccumulation of all five ERF-VII proteins. However, the impact of the *prt6* mutation on mRNA accumulation of *ERF-VII* genes remains unknown. This study revealed that the *prt6* mutation increases mRNA levels of all *ERF-VII* genes under salinity, along with the dramatic induction of *ADH* mRNA, a downstream target of ERF-VII transcription factors (Figure 8). The expression patterns of HRE-type and RAP2-type *ERF-VIIs* were apparently distinct, reflecting the notion that these two types of ERF-VIIs function differently in the regulation of hypoxia-responsive gene expression and seed germination [14,58,59]. The mechanisms underlying overaccumulation of *ERF-VII* mRNAs in the *prt6* mutant are unknown, but this process must assist the further synthesis of ERF-VII proteins.

The functional importance of *ERF-VII* genes and proteins in salinity tolerance has been previously reported. For example, transgenic and mutant analysis revealed that *HRE2* is a positive regulator for salinity tolerance in *Arabidopsis* [60]. Vicente et al. [26] demonstrated that salinity tolerance conferred by the *prt6* mutation is attributable to increased accumulation of ERF-VII proteins because the resilience was reverted in the *prt6erfVII* sextuple mutant that lacks *PRT6* and all five *ERF-VII* genes. This report also suggested that high salt promotes the stabilization of ERF-VII proteins through a reduced synthesis of nitric oxide, an essential molecule for the targeted degradation of ERF-VIIs via the PRT6/N-degron pathway. These results emphasize that the PRT6/N-degron pathway regulation of ERF-VIIs plays a vital role in salinity tolerance in Arabidopsis. Further genetic and molecular analyses will determine the impact of this pathway in the regulation of ABA-dependent and independent processes and hormone response pathways under salinity stress.

**Figure 9:**
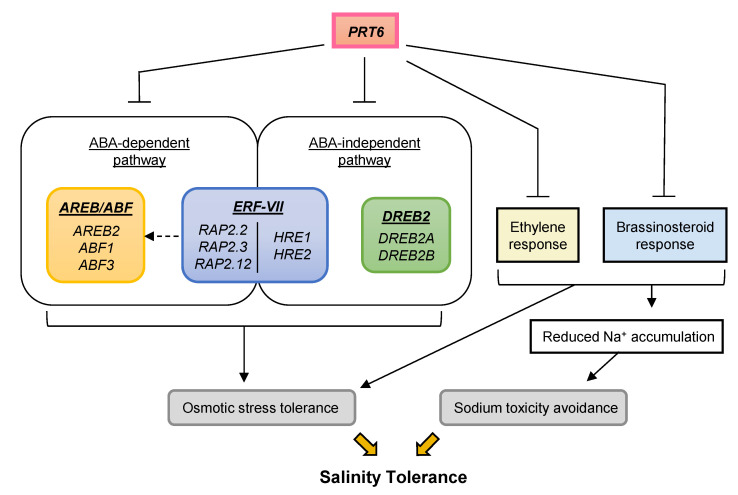
Model for salinity-tolerance mechanisms that are negatively regulated by the PRT6/N-degron pathway. A dashed line indicates a hypothetical relationship.

We propose a model for salinity-tolerance mechanisms that are negatively regulated by the PRT6/N-degron pathway (Figure 9). When exposed to high salt, plants are damaged mainly due to salt-induced osmotic stress and sodium toxicity. The present study demonstrated that *PRT6* restricts signaling processes associated with adaptation to both osmotic stress and sodium toxicity. For example, *PRT6* downregulates mRNA accumulation of *AREB*/*ABFs* and *DREB2s*, well-characterized transcription factors involved in the ABA-dependent and independent pathways of osmotic stress tolerance, respectively. *PRT6* also dampens responsiveness to ethylene and BR, key hormones that enhance the expression of stress-responsive genes such as dehydrins and restrict root Na^+^ uptake and root-to-shoot Na^+^ transport. In this model, we propose that *RAP2*-type *ERF-VIIs* serve as members of the ABA-dependent pathway, whereas *HRE*-type *ERF-VIIs* function in the ABA-independent pathway due to the following reasons: (1) All *RAP2*-type *ERF-VII* genes are ABA-responsive [43]. (2) The three RAP2-type ERF-VIIs directly regulate the expression of *ABI5*, a downstream transcription factor in the ABA signaling pathway [14]. (3) Inducible expression of each of the three *RAP2*-type *ERF-VIIs* increases sensitivity to ABA [43]. (4) Both *HRE*-type *ERF-VII* genes are not ABA-inducible [43,61]. (5) HRE2 activates the expression of reporter genes by direct binding to the DRE/CRT motif, a *cis*-acting element in the ABA-independent pathway [62,63]. These data demonstrate that *RAP2* and *HRE*-type *ERF-VIIs* belong to the ABA-dependent and independent pathways of osmotic/salinity stress tolerance, respectively. Based on the evidence (2), it is predicted that RAP2-type ERF-VIIs directly modulate the expression of *AREB/ABFs*, other downstream transcription factors in the ABA signaling pathway. Taken together, these results have revealed that the PRT6/N-degron pathway acts as a negative regulator of salinity tolerance in *Arabidopsis* through modulation of transcriptional and hormonal responses to the stress. Many commercially important plants can encounter salinity and flooding sequentially or concurrently, reducing crop yield and quality under changing climates. Guided manipulation of the PRT6/N-degron pathway could form a promising approach to generate new crop varieties with improved tolerance to both salinity and flooding.

## 4. Materials and Methods

### 4.1. Plant Materials and Growth Conditions

Arabidopsis (*Arabidopsis thaliana* L.) Columbia-0 [wild-type (WT)] and its T-DNA insertion mutant, *prt6-1*, were obtained from the Arabidopsis Biological Resource Center at Ohio State University. The *prt6-1* knockout mutant has been described previously [10,11,12,64]. These genotypes were propagated simultaneously under the same growth conditions (23 °C, 50% relative humidity, 16 h day/8 h dark, 120 µmol photons m^−2^ s^−1^). Seeds were sterilized with 2% (*w*/*v*) sodium hypochlorite and 0.1% (*v*/*v*) Tween-20 for 12 min and then rinsed thoroughly using deionized water. The sterilized seeds were immersed in water at 4 °C for 4 d for synchronized germination.

### 4.2. Salinity Stress and Hormone Treatments

All stress and hormone treatments were replicated in at least three independent biological experiments under the growth conditions mentioned above. For germination tests, seeds were incubated on half-strength MS media containing 0, 50, 100, or 150 mM NaCl for up to 5 d. Seedlings with more than 1 mm roots were counted as germinated. To visualize root growth under salinity, root bending assays [27,28] were performed. Four-day-old seedlings were transferred on half-strength MS media containing 0 or 150 mM NaCl and grown upside down for 4 d. To quantify root growth under salinity, the root tip of the main root in each seedling was marked after transplanting of 4-day-old seedlings onto salt-containing media (0–150 mM NaCl). Following 4 d of vertical incubation, the length of root growth was measured. For seedling viability tests, 4-day-old seedling grown under non-stress conditions were transferred into half-strength MS media containing 0 or 200 mM NaCl and incubated for 4 d. For adult plant viability tests, 4-day-old seedlings were grown in soil-containing pots for 21 d. These pots were placed in a tray containing 0 or 200 mM NaCl for 12 d.

Hormone treatments were performed by transferring 10-day-old seedlings on half-strength MS media supplemented with 1-aminocyclopropane-1-carboxylic acid (ACC; 0, 1, or 100 µM) or 24-epibrassinolide (eBL; 0, 0.01, or 1 µM in 0.02% (*v*/*v*) ethanol) and incubating them for 6 h.

### 4.3. Chlorophyll and Anthocyanin Assays

Chlorophyll and anthocyanin contents were determined using the methods of Porra [65] and Rabino and Mancinelli [66], respectively. Chlorophyll was extracted from 50 mg of homogenized fresh tissues in 3 mL of 100% methanol on ice. Following centrifugation at 4 °C for 20 min at 21,000 g, the absorbance of the supernatant was measured at 652.0 and 665.2 nm with a UV-Vis spectrophotometer. Anthocyanin was extracted from 200 mg of homogenized fresh tissues by shaking the tissues in acidic methanol extraction buffer [99% (*v*/*v*) methanol +1% (*v*/*v*) HCl] for 16 h at 4 °C. After extraction, 0.4 mL of water and 0.4 mL of chloroform were added. Following centrifugation at 4 °C for 2 min at 21,000 g, the absorbance of the supernatant was measured at 530 and 657 nm.

### 4.4. Na^+^ and K^+^ Content Analysis

Sodium and potassium ion concentrations were quantified using the method of Rus et al. [67]. Four-day-old seedlings were grown in pots containing Turface MVP (Turface Athletics, Buffalo Grove, IL, USA) for 21 d. These pots were placed in a tray containing 0 or 200 mM NaCl, and shoot and root tissues were harvested on the specified days. The harvested tissues were washed using deionized water and dried at 65 °C for 2 d. Tissues (100 mg dry weight) were then homogenized in 20 mL of 0.1 N HNO_3_ for 30 min. Following filtration with quantitative analysis-grade filter paper, the extract was subjected to Na^+^ and K^+^ analysis using an inductively coupled plasma atomic emission spectrometer (ICP-AES).

### 4.5. Hormone Sensitivity Evaluation

For ethylene response analysis, seeds were incubated on half-strength MS media containing 0, 1, 10, or 100 µM ACC in the dark for 5 d. For brassinosteroid response analysis, seeds were grown on half-strength MS media containing 0, 0.01, 0.1, or 1 µM eBL in the light (120 µmol m^−2^ s^−1^) for 5 d. After incubation, hypocotyl and root length was measured using Image J software [68].

### 4.6. Quantitative RT-PCR Analysis

Total RNA was extracted from 100 mg (fresh weight) of seeding tissue using RNeasy Plant Mini kit (Qiagen, Hilden, Germany). Genomic DNA was eliminated by the on-column digestion method described by the manufacturer’s protocol. cDNA was synthesized from 2 µg of total RNA as described by Fukao et al. [69]. Real-time RT-PCR was conducted in a 15 µL reaction using iTaq Universal SYBR Green Supermix (Bio-Rad, Hercules, CA, USA) in the CFX Connect real-time PCR detection system (Bio-Rad). Amplification specificity was validated by melt-curve analysis at the end of each PCR experiment. Relative transcript abundance was determined using the comparative cycle threshold method [70]. Primer sequences used for this analysis are listed in Table S1. *UBQ10* (AT4G05320) and *18s RNA* (AT2G01010) genes were used as internal controls. qRT-PCR was repeated three times using tissues generated from three independent plant culture under identical growth conditions.

## Supplementary Materials

The following are available online at https://www.mdpi.com/2223-7747/9/11/1415/s1, Table S1: Primer sequences used for quantitative RT-PCR.
