# Supplementary material for: The Central Negative Regulator of Flooding Tolerance, the PROTEOLYSIS 6 Branch of the N-degron Pathway, Adversely Modulates Salinity Tolerance in Arabidopsis
Source: Plants (Basel). 2020 Oct 23;9(11):1415. doi: 10.3390/plants9111415 (PMC7690746; doi:10.3390/plants9111415)
Supplement: Supplementary file 1 [file plants-09-01415-s001.pdf]

Table S1. Primer sequences used for quantitative RT-PCR.

Gene name	Gene ID	Forward primer (5' ==> 3')	Reverse primer (5' ==> 3')	References
ABF1	AT1G49720	TGCAGAAGAAACAGGCTGAA	GACCGGTAAGGGTTCTTCTCA	Yoshida et al., 2015
ABF3	AT4G34000	CGTTCTCAACCTGCAACACA	TCATAGGATGGTTATGAATTCCAAG	Yoshida et al., 2015
AREB1	AT1G45249	GAGAGAAGGCAAAGGAGAATGA	CTTCAAGCTCCACGGTGTAAG	Yoshida et al., 2015
AREB2	AT3G19290	GCAACCTGGTGCTGGTATCC	TGTTCTAGGCAACGTCAACGA	Yoshida et al., 2015
DREB2A	AT5G05410	CAGTGTTGCCAACGGTTCAT	AAACGGAGGTATTCCGTAGTTGAG	Sakuma et al., 2006
DREB2B	AT3G11020	CAGGCCGGACAGTTGCA	CCCAATACTGCTGCTCAAACTCTA	Sakuma et al., 2006
RD29A	AT5G52310	TGGATCTGAAGAACGAATCTGATATC	GGTCTTCCCTTCGCCAGAA	Sakuma et al., 2006
RD29B	AT5G52300	GCAAGCAGAAGAACCAATCA	CTTTGGATGCTCCCTTCTCA	Yoshida et al., 2015
RAB18	AT1G43890	TCGGTCGTTGTATTGTGCTTTTT	CCAGATGCTCATTACACACTCATG	Yoshida et al., 2015
SOS1	AT2G01980	TGGAGGAAGCGACCGATTC	CGATAACGAGAAGAGCGACAG	Cheng et al., 2016
HKT1	AT4G10310	TCTGGTCTCATCTGGCTCCTAATC	CACCGTCACTCCAAGAAGAACAC	Cheng et al., 2016
HRE1	AT1G72360	GGCCTCTGCCTTATCCCTCTGT	GCGTAAACCCGTCTCAGTGAGTG	Licausi et al., 2010
HRE2	AT2G47520	GAAGCGTAAACCCGTCTCAGT	TTTGCTCGGGCACGAATCT	Licausi et al., 2010
RAP2.2	AT3G14230	AATCCGTGGCACGAAAGC	GGATACGACGCTAGGGTTCTTCT	Papdi et al., 2015
RAP2.3	AT3G16770	CGTCATCGCCACGATCAA	TGAGAGACAACGCAGACCTTCTT	Papdi et al., 2015
RAP2.12	AT1G53910	CGCTGCGGAAGGTTCAGTT	CAGCCCATTTTCCCCAAGGA	Paul et al., 2016
ADH1	AT1G77120	TATTCGATGCAAAGCTGCTGTG	CGAACTTCGTGTTTCTGCGGT	Paul et al., 2016
PDF1.2	AT5G44420	CACCCTTATCTTCGCTGCTCTT	GCCGGTGCGTCGAAAG	Van der Does et al., 2013
ACO2	AT1G62380	CCTCCTCTCAACCACTCTATTGTC	CATCTTCATAAACATAAACACCCCACAC	Dong et al., 2015
ERF1	AT3G23240	ATTCTTTCTCATCCTCTTCTTCT	CGAATCTCTTATCTCCGCCG	Mao et al., 2016
BAS1	AT2G26710	CCAAGGACCATGTCGTTAAGC	CCTGAAGTATAGCAAGATTCTGACC	Schneider et al., 2012
PAE8	AT4G19420	GGGCATCATTCACAGGAGAT	CAGCAGAACAGCCAGACAAA	Xie et al., 2015
XTH33	AT1G10550	ATACCCTCATTTGGAACTCCCACC	CACTTAACTCCACGTCAGCAACG	Dong et al., 2014
Saur_AC1	AT4G38850	CAGAAGAAGAAGAGATATGTGGTG	GTATTGTTAAGCCGCCCATTG	Zhu et al., 2013
18S RNA	AT2G01010	AAACGGCTACCACATCCAAG	CCTCCAATGGATCCTCGTTA	Yoshida et al., 2015
UBQ10	AT4G05320	AGATCCAGGACAAGGAAGGTATTC	CGCAGGACCAAGTGAAGAGTAG	Lin et al., 2013

References

Dong, J., Tang, D., Gao, Z., Yu, R., Li, K., He, H., et al. (2014) Arabidopsis DE-ETIOLATED1 represses photomorphogenesis by positively regulating phytochrome-interacting factors in the dark. *Plant Cell* 26: 3630-3645.

Dong, Z., Yu, Y., Li, S., Wang, J., Tang, S. and Huang, R. (2016) Abscisic Acid Antagonizes Ethylene Production through the ABI4-Mediated Transcriptional Repression of ACS4 and ACS8 in Arabidopsis. *Mol. Plant* 9: 126-135.

Li, P.C., Huang, J.G., Yu, S.W., Li, Y.Y., Sun, P., Wu, C.A., et al. (2016) Arabidopsis YL1/BPG2 Is Involved in Seedling Shoot Response to Salt Stress through ABI4. *Sci. Rep.* 6: 30163.

Licausi, F., van Dongen, J.T., Giuntoli, B., Novi, G., Santaniello, A., Geigenberger, P., et al. (2010) HRE1 and HRE2, two hypoxia-inducible ethylene response factors, affect anaerobic responses in Arabidopsis thaliana. *Plant J.* 62: 302-315.

Lin, W., Lu, D., Gao, X., Jiang, S., Ma, X., Wang, Z., et al. (2013) Inverse modulation of plant immune and brassinosteroid signaling pathways by the receptor-like cytoplasmic kinase BIK1. *Proc. Natl. Acad. Sci. USA* 110: 12114-12119.

Mao, J.L., Miao, Z.Q., Wang, Z., Yu, L.H., Cai, X.T. and Xiang, C.B. (2016) Arabidopsis ERF1 Mediates Cross-Talk between Ethylene and Auxin Biosynthesis during Primary Root Elongation by Regulating ASA1 Expression. *PLoS Genet.* 12: e1005760.

Papdi, C., Perez-Salamo, I., Joseph, M.P., Giuntoli, B., Bogre, L., Koncz, C., et al. (2015) The low oxygen, oxidative and osmotic stress responses synergistically act through the ethylene response factor VII genes RAP2.12, RAP2.2 and RAP2.3. *Plant J.* 82: 772-784.

Paul, M.V., Iyer, S., Amerhauser, C., Lehmann, M., van Dongen, J.T. and Geigenberger, P. (2016) Oxygen Sensing via the Ethylene Response Transcription Factor RAP2.12 Affects Plant Metabolism and Performance under Both Normoxia and Hypoxia. *Plant Physiol.* 172: 141-153.

Sakuma, Y., Maruyama, K., Osakabe, Y., Qin, F., Seki, M., Shinozaki, K., et al. (2006) Functional analysis of an Arabidopsis transcription factor, DREB2A, involved in drought-responsive gene expression. *Plant Cell* 18: 1292-1309.

Schneider, K., Breuer, C., Kawamura, A., Jikumaru, Y., Hanada, A., Fujioka, S., et al. (2012) Arabidopsis PIZZA has the capacity to acylate brassinosteroids. *PLoS One* 7: e46805.

Van der Does, D., Leon-Reyes, A., Koornneef, A., Van Verk, M.C., Rodenburg, N., Pauwels, L., et al. (2013) Salicylic acid suppresses jasmonic acid signaling downstream of SCFCOI1-JAZ by targeting GCC promoter motifs via transcription factor ORA59. *Plant Cell* 25: 744-761.

Xie, Y., Straub, D., Eguen, T., Brandt, R., Stahl, M., Martinez-Garcia, J.F., et al. (2015) Meta-Analysis of Arabidopsis KANADI1 Direct Target Genes Identifies a Basic Growth-Promoting Module Acting Upstream of Hormonal Signaling Pathways. *Plant Physiol.* 169: 1240-1253.

Yoshida, T., Fujita, Y., Maruyama, K., Mogami, J., Todaka, D., Shinozaki, K., et al. (2015) Four Arabidopsis AREB/ABF transcription factors function predominantly in gene expression downstream of SnRK2 kinases in abscisic acid signalling in response to osmotic stress. *Plant Cell Environ.* 38: 35-49.

Zhu, W., Wang, H., Fujioka, S., Zhou, T., Tian, H., Tian, W., et al. (2013) Homeostasis of brassinosteroids regulated by DRL1, a putative acyltransferase in Arabidopsis. *Mol. Plant* 6: 546-558.
